# Drag reduction study of a microfiber-coated cylinder

**DOI:** 10.1038/s41598-022-19302-5

**Published:** 2022-09-02

**Authors:** Mitsugu Hasegawa, Yi-Chung Chen, Hirotaka Sakaue

**Affiliations:** Department of Aerospace and Mechanical Engineering, University of Notre Dame, Notre Dame, IN 46556 USA

**Keywords:** Environmental impact, Mechanical engineering, Civil engineering

## Abstract

Drag reduction for a bluff body is imperative in a time of increasing awareness of the environmental impact and sustainability of air travel. Microfiber coating has demonstrated its ability to reduce drag on a bluff body. This was done by applying strips of the coating to a cylinder. To widen the application range of the microfiber coating, a fully microfiber-coated cylinder is studied as it has no directionality relative to incoming flow. It is hypothesized that a large coating coverage will cause a reduction in drag dependent on the Reynolds number *Re*. The fully microfiber-coated cylinder is studied in a wind tunnel and the drag coefficient is determined at a range of *Re* in the subcritical-flow regime. It is found that the drag coefficient of the microfiber-coated cylinder is a function of *Re*, and the critical Reynolds number, where the maximum drag reduction occurs, is lower for a microfiber-coated cylinder compared to that of a conventional smooth-surface cylinder.

## Introduction

Drag reduction for an energy-efficient bluff-body system is a major focus in fluid dynamics^1,2^. It has impactful applications in improving fuel efficiency on vehicles, which is critical in a time of rising concern over the adverse environmental impact from vehicle emissions^3–5^. For a bluff body, pressure drag is generated when an adverse pressure gradient causes the flow over the body to separate, but this drag can be mitigated by keeping the flow attached^6,7^. A cylinder is commonly used as a representative geometry for bluff bodies and has been studied extensively^8–10^. Therefore, there are numerous efforts to develop a drag-reduction device on a cylinder^11,12^. Examples of such flow-control devices include surface roughness alterations^13–15^, cylindrical rods^16–23^, dimples^24,25^, vortex generators^26,27^, permeable surfaces^28–30^, and textile surfaces^31,32^.

A hair-like structure has the flexibility to produce both passive and adaptive effects on the flow. Several numerical studies have shown that spontaneous symmetry breaking of a flexible filament attached to the rear of a cylinder reduces drag and produces lift^33–36^. While these studies focus on development of theoretical models using a numerical approach, Hasegawa and Sakaue experimentally introduced microfiber coating as a flexible hair-like structure for drag-reduction^37,38^. Their studies have shown that a cylinder with strips of microfiber coating achieved drag reduction if the strips were placed between 10° and 60° and between 100° and 140° from the front stagnation location^38^. The former region is a windward location prior to flow separation, and the latter region is a leeward location after the separation at a fixed Reynolds number *Re* of 6*.*1 × 10^4^. Fibers less than 2% of a cylinder diameter in length achieved a higher drag reduction when placed on the windward side. In contrast, fibers with a length greater than 3% of a cylinder diameter resulted in a higher drag reduction on the leeward side. Strips of the coating with a length of 1% cylinder diameter placed symmetrically on the cylinder relative to the flow direction yielded the highest drag reduction of 51% at 40°^39^. Longer fibers 8% of a cylinder diameter in length placed on the leeward side yielded a maximum drag reduction of 16% at 110°^40^. Based on the configurations in previous studies, a hypothesis arises that a shorter fiber and a wider coating coverage on the windward side could best enhance the drag reduction. To apply the microfiber coating to various fluid dynamics problems, it is desirable to remove the directionality of the coating by fully covering the coating over a cylinder surface. However, it is unclear whether the fully covered coating would reduce drag. Because cylinder drag is a function of *Re*^13,41^, another hypothesis arises that the drag reduction performance of the fully microfiber-coated cylinder will also be a function of *Re*.

The objective of this study is to evaluate these hypotheses by determining the drag coefficient of the fully microfiber-coated cylinder for a range of *Re* in the subcritical-flow regime. It will also demonstrate the drag reduction performance of a fully microfiber-coated cylinder, which can be compared to the aforementioned configurations.

## Experimental setup and method

### Wind tunnel facility and cylinder model

The experiment was conducted using the subsonic wind tunnel in Hessert Laboratory at the University of Notre Dame. The test section of the wind tunnel has a 0.61 m square cross-section and a length of 1.83 m. The tunnel is an open return design, which drafts airflow into the test section from the lab space. The wind tunnel includes a contraction section with a ratio of 20:1 to uniformly distribute airflow. A series of 12 honeycomb patterned screens is installed at the inlet of wind tunnel to achieve a turbulence level of 0.1% in freestream.

A circular cylinder with and without microfiber coating was placed in a cross flow to produce a flow field at the test section. The circular cylinder used was machined out of a solid plastic. The cylinder had a diameter *D* of 50 mm, a spanwise length *L* of 610 mm, and aspect ratio *L/D* of 12. The blockage ratio of the cylinder to the test section was 8%. The *Re* based on the cylinder diameter was varied from 2 × 10^4^ to 10 × 10^4^, which were within the subcritical-flow regime^10,42^. The *Re* based on the cylinder diameter was determined from the following equation:

1 $$Re=\frac{U_{\infty }D}{\nu }$$

where *U*_∞_ is the freestream airspeed and *ν* is the kinematic viscosity of air.

**Figure 1:**
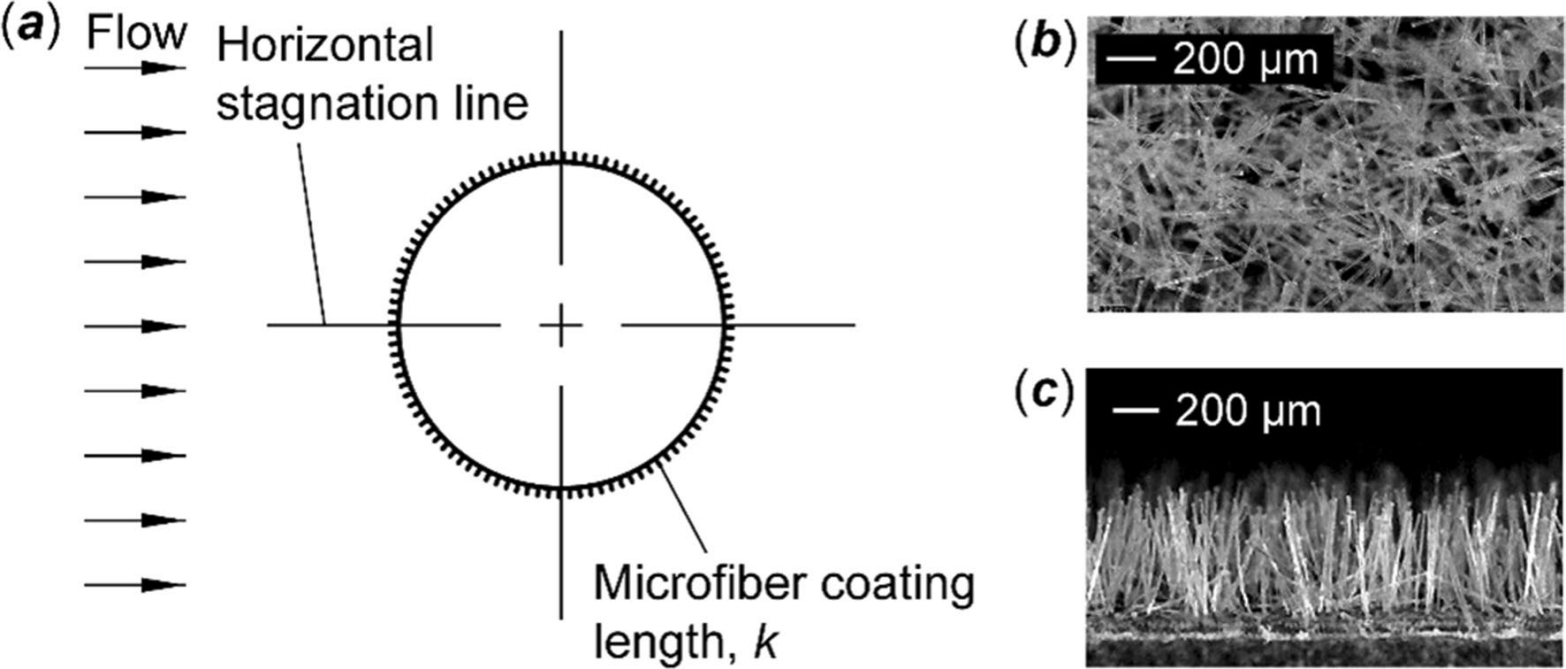
A schematic of the fully microfiber-coated cylinder. (**a**) Coating configuration on the cylinder model. The fiber length *k* was 0.5 mm, which was 1% of the cylinder diameter. (**b**) A microscope image of fabricated microfiber coating seen from top. (**c**) A microscope image of fabricated microfiber coating seen from lateral.

The cylinder was fully covered by the microfiber coating, as illustrated in Fig. 1a. The fiber length *k* was 0.5 mm based on the previous study^38,39^. With respect to the cylinder diameter, the fiber has a ratio of the length to cylinder diameter *k/D* of 1%. Figure 1b and c show microscopic images of the microfiber coating. The diameter of the fiber was about 14 microns. By counting the number of microfibers per unit area on the microscope images, the mean surface density of the fiber was found to be 121 fibers/mm^2^. The microfiber coating was made from Nylon 6/6, Poly (hexamethylene adipamide, Campbell Coutts Ltd., Eastleigh, U.K.). The Young’s modulus was estimated to be around 26–46 [cN/dtex]. The microfiber coating was fabricated by applying the fibers on a 50 µm thick layer of epoxy coated on the cylinder surface using an electrostatic flocking method^43^.

### Wake profile measurement

**Figure 2:**
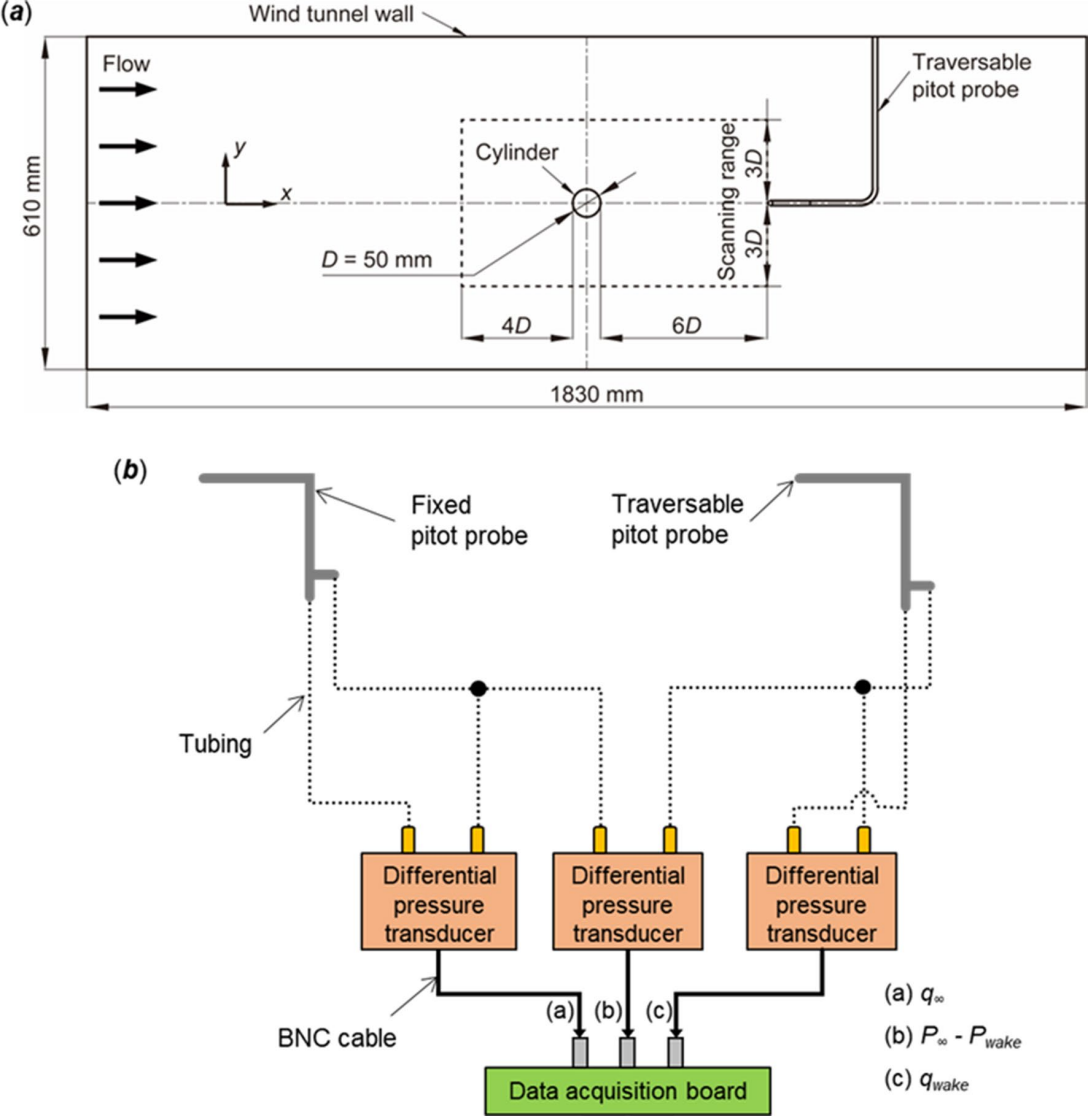
Schematic of the wake profile measurement. (**a**) Configuration of cylinder model and pitot probes in the wind tunnel. (**b**) Setup of differential pressure measurement using pitot probes.

The cylinder wake profile was measured by a vertically traversing Pitot probe at a location 6*D* downstream of the trailing edge of the cylinder. A set up of the wake profile measurement is schematically shown in Fig. 2a and b. The wake was scanned at a range of ± 3*D* from the horizontal centerline of the cylinder. Throughout the scanning range, a total of 25 data points were collected, with an interval of 12.7 mm between each point. A step motor traverse system (PDO3540, Applied Motion Products, Watsonville, CA, USA) was used to control the scan. The freestream flow was also measured by a fixed Pitot probe at a location 4*D* upstream of the leading edge of the cylinder. The dynamic pressure in the cylinder wake was measured to determine the wake velocity profile. The pressure difference between the upstream *P*_∞_ and the wake *P*_*wake*_ were also measured to calculate the drag described in later sections. The freestream dynamic pressure *q*_∞_ was measured to determine the freestream speed. Differential pressure transducers (Model 239, Setra Systems, Boxborough, MA, USA), which have a full-scale range of 0 to 2.5 inches of water and accuracy of ± 0.14% of its full-scale reading, were used for pressure measurements. A data acquisition board (DT9836, Data Translation, Marlborough, MA, USA) collected the output signals of the pressure transducer. Each pressure measurement was sampled at 1 kHz for 10 s. The collected dataset was time averaged.

### Flow visualization

A smoke flow visualization around the cylinder was performed to understand the flow influenced by microfiber coating. A point of flow separation was determined using the acquired image from the flow visualization. Fog streak lines along the center plane of the wind tunnel test section were generated by injecting seeding particles from the upstream of the wind tunnel. The seeding particles of droplets (TOPAS, DEHS Aerosol Liquid for Atomizer) were generated by a particle seeder (TSI, SIX-JET ATOMIZER9306) and were of less than 1 μm in diameter. The streamed flow was illuminated by a laser sheet provided by a blue laser (NECSEL, Blue 445-10 W) with a cylindrical lens. The fog streak lines were captured by a high-speed camera (Photron, FASTCAM AX100) with a frame rate of 3600 frame per second.

### Drag estimation

The drag per unit span of the cylinder *f* was obtained by the change in momentum in the streamwise direction inside a two-dimensional control volume^44^:

2 $$f=\rho {\int }_{y=-3D}^{y=+3D}{u}_{wake}({U}_{\infty }-{u}_{wake})dy+{\int }_{y=-3D}^{y=+3D}({P}_{\infty }-{P}_{wake})dy$$

where *ρ* is the density of air, *U*_∞_ is the uniform velocity upstream of the cylinder in the freestream, *u*_*wake*_ is the velocity profile downstream of the cylinder, *P*_∞_ is the freestream pressure upstream of the cylinder, and *P*_*wake*_ is the pressure in the wake downstream of the cylinder, respectively. Here, the control volume ranged from − 3*D* to 3*D* in the *y*-direction, and from − 4*D* to 6*D* in the *x*-direction, respectively, as shown by the dashed box in Fig. 2. The change in momentum can be calculated from the pressures and the velocity profiles on the upstream and downstream planar control volume surfaces. The velocity profiles and the change in static pressures on those surfaces were integrated to determine *f*.

The drag coefficient per unit span of the cylinder *c*_*d*_ can then be calculated by:

3 $${c}_{d}=\frac{f}{D\cdot {q}_{\infty }}$$

To discuss the effects on the microfiber coating to the drag, the Drag Impact *DI* was defined:

4 $$DI (\%)=\frac{{c}_{d\_coating}-{c}_{d\_baseline}}{{c}_{d\_baseline}}\times 100$$

where *c*_*d_coating*_ and *c*_*d_baseline*_ are the drag coefficients of the fully microfiber-coated cylinder and the baseline cylinder, respectively. These were determined using Eqs. (2) and (3) with respective pressures and velocity profiles. The *DI* directly compares the change in *c*_*d*_ at a given *Re*; a negative *DI* indicates a drag reduction by the microfiber coating, while a positive *DI* indicates a drag increase.

## Results and discussion

### Flow visualization

**Figure 3:**
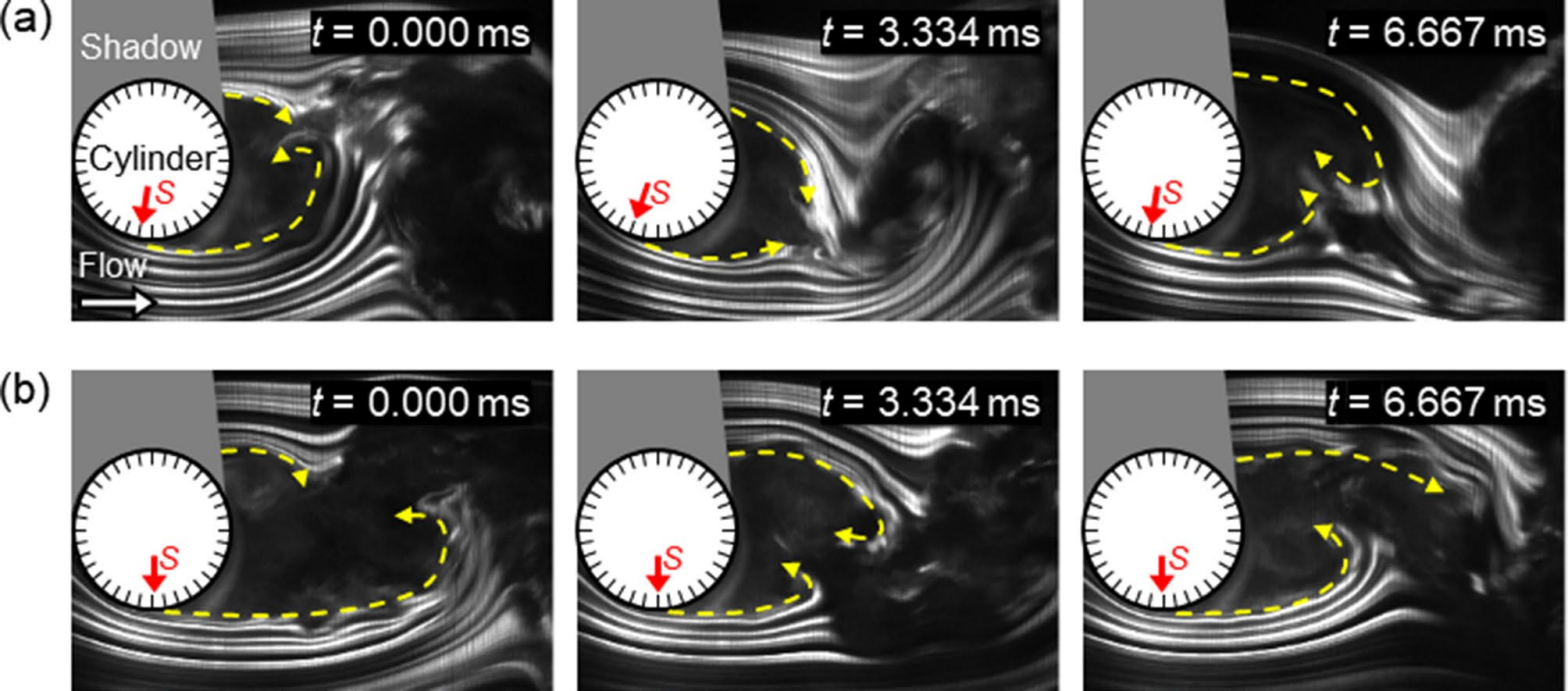
Time series of streaklines around the cylinder captured using smoke flow visualization. The *Re* based on the cylinder diameter was 6.6 × 10^4^. Symbol, *S*, indicates the point of flow separation. Dashed line with arrows indicates separating shear layers. Comparison of the point of flow separation for (**a**) baseline, and (**b**) microfiber coating.

Figure 3a and b show a time series of streaklines around the cylinder for the baseline and microfiber-coated cylinder, respectively. The *Re* based on the cylinder diameter was 6.6 × 10^4^. For the baseline shown in Fig. 3a, periodic changes in the streaklines were observed due to vortex shedding, and flow separation occurred at around 80°. This behavior is expected based on the flow pattern for a smooth cylinder in a subcritical flow regime^42^, and it is estimated that the baseline had a laminar boundary layer based on the Reynolds number in this study. For the microfiber coating shown in Fig. 3b, the point of flow separation was delayed compared to that of the baseline. The point of flow separation was extended to around 90°, and the wake width, which was shown between the separating shear layers, was narrowed downstream of the cylinder.

Flow separation occurs on the cylinder at a larger angle relative to the leading-edge stagnation point for the microfiber coated cylinder than for the baseline case. This is because the microfiber coating, due to its interaction with the flow, causes a laminar to turbulent boundary layer transition over the cylinder. The increased mixing of the turbulent boundary layer relative to the laminar boundary layer means that flow stays attached over a greater extent of the cylinder. This is visible in Fig. 3a and b, which show that the addition of the microfiber coating caused the separation point to the flow to shift towards the trailing edge of the cylinder.

### Wake profile

**Figure 4:**
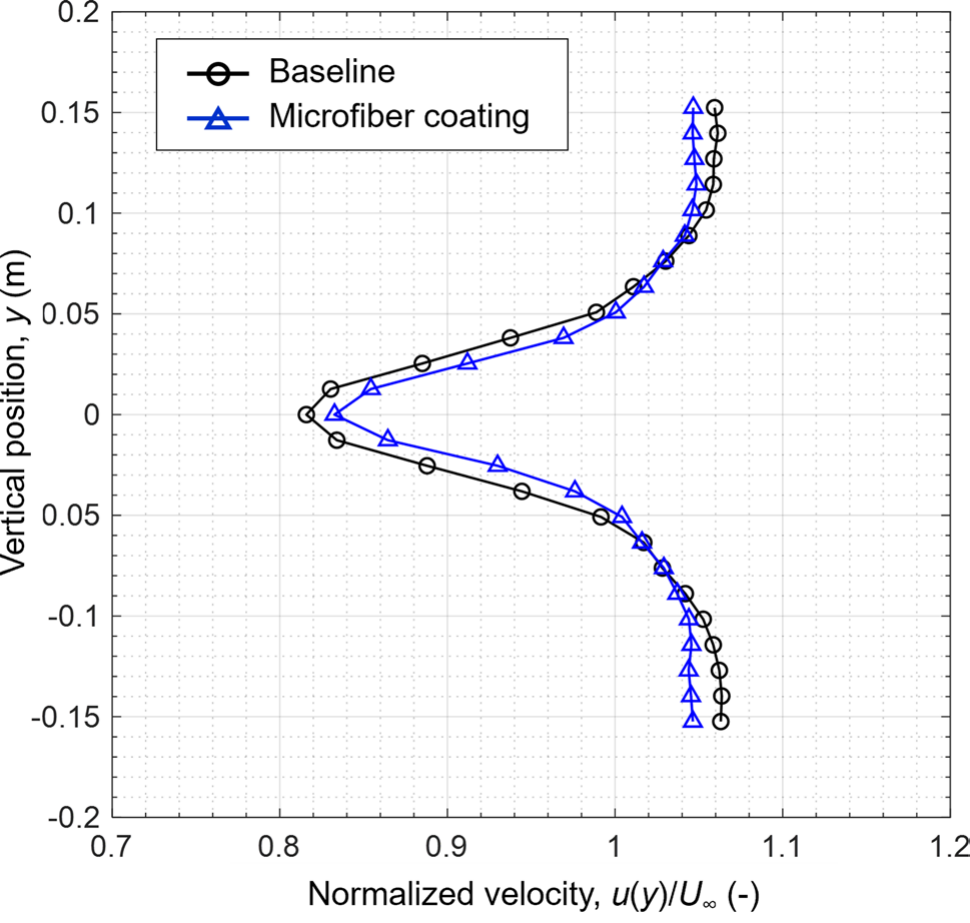
Normalized velocity profile in the wake of different cylinder configurations. The *Re* based on the cylinder diameter was 6.6 × 10^4^.

Figure 4 shows wake velocity profiles normalized by the freestream velocities for the baseline cylinder and for the fully microfiber-coated cylinder, respectively. The one with the microfiber coating could reduce the velocity deficit, which indicates that the wake region downstream of the cylinder was reduced. Based on Eq. (2), the reduction in the velocity deficit results in drag reduction. Since the wake velocity profile is normalized by the upstream freestream velocity, the profile was greater than unity outside of the wake. For the microfiber-coated cylinder, the velocity outside of the wake was less than that of the baseline cylinder. This is because the baseline cylinder had a larger wake with a greater velocity deficit, resulting in an increase of the velocity outside of the wake to conserve mass.

### Drag coefficient

**Figure 5:**
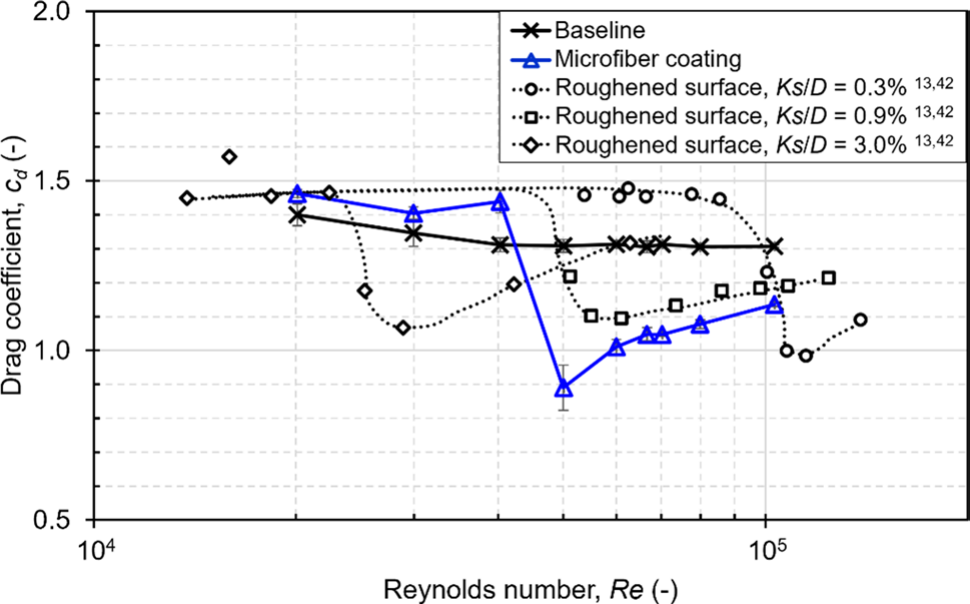
Drag coefficient *c*_*d*_ of the fully microfiber-coated cylinder compared to a baseline cylinder and a cylinder with a roughened surface for a range of Reynolds Numbers *Re*.

From equations. (2) and (3), the *c*_*d*_ of the baseline cylinder and the microfiber-coated cylinder was obtained. The solid and wake blockage around a cylinder increased the dynamic pressure causing overestimation of the drag coefficients^45^. A blockage correction method by Maskell was applied to deal with the blockage effect^46^. The measurement of the wake profile was repeated 3 times. The *c*_*d*_ calculated for the 3 experiments was averaged to provide the mean and standard deviation for drag coefficient. The *c*_*d*_ curves in Fig. 5 are plotted then as the mean values, with error bars based on the standard deviation of the 3 experiments. The baseline *c*_*d*_ ranged from 1.3 to 1.4 depending on *Re*, which was similar to *c*_*d*_ obtained by Achenbach^13^. 1.3 to 1.4 for the baseline *c*_*d*_ is higher than the 1.0 to 1.2 often shown in literatures^42^. This is due to the uncertainty of wake measurements based on pitot probes^47^.

From Fig. 5, it is shown that the *c*_*d*_ of the microfiber-coated cylinder is a function of *Re*, and had a higher drag compared to the baseline cylinder in the lower half of the tested *Re,* namely at *Re* between 2 × 10^4^ and 4 × 10^4^ . At *Re* ≈ 5 × 10^4^, the drag crisis was seen for the microfiber-coated cylinder. The critical *Re* for a cylinder with a smooth surface, such as the baseline cylinder, is around 3 × 10^5^^13^. By applying the microfiber coating entirely over a cylinder surface, the critical *Re* was shown at a lower *Re*. The drag reduction due to the microfiber coating was the result of the shift of the critical *Re* to a smaller *Re*.

The drag coefficient of microfiber-coated cylinder can be compared with that of a cylinder with a roughened surface as shown in Fig. 5^13,42^. Both the microfiber coating and roughened surface introduce three-dimensional roughness which enhances boundary layer transition. The major difference between the microfiber coating and the roughened surface is that the former is a flexible device, and the latter is a rigid device. The cylinder with roughness height, *Ks*, equal to 0.9% of cylinder diameter, which is close to the microfiber height used in the present study, showed a similar shift in the critical *Re* for that of the cylinder with microfiber coating ^13,42^. However, the critical *Re* of the microfiber coating is lower than that of the roughened surface. The critical *Re* for the microfiber-coated and roughened cylinder were 5 × 10^4^, and between 5.5 × 10^4^ to 6 × 10^4^ respectively ^13,42^.

### Drag impact

**Figure 6:**
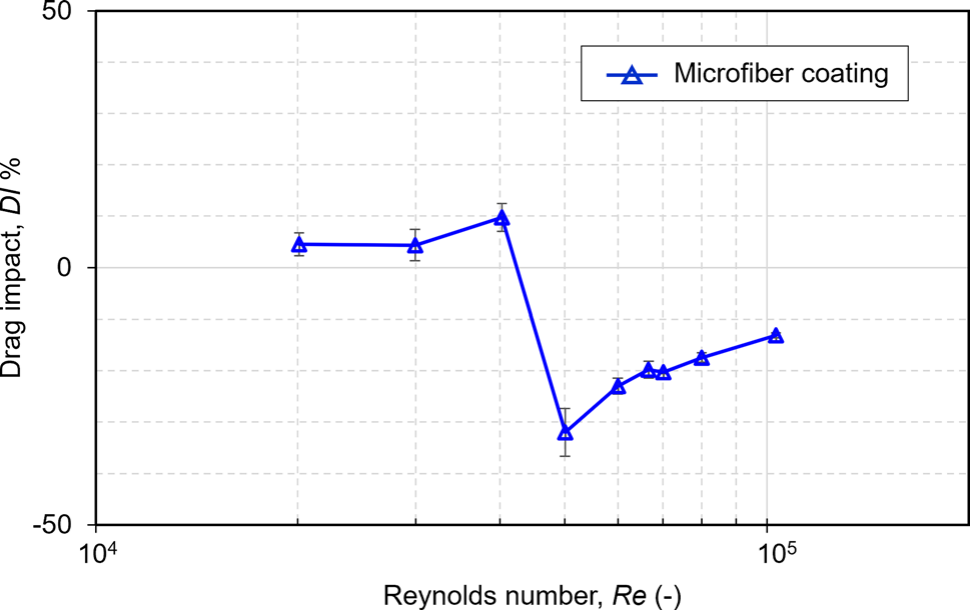
Drag impact *DI* related to the Reynolds Number *Re*.

Figure 6 shows the *DI* related to *Re* to directly compare the drag of the baseline and microfiber-coated cylinders at same *Re*. The *DI* did not show a monotonic trend. A drag increase, indicated by a positive *DI*, of around 5% existed for *Re* between 2*.*0 × 10^4^ and 3*.*0 × 10^4^, and around 10% at a *Re* of 4*.*0 × 10^4^. Above a *Re* of 4.5 ×  − 10^4^, the *DI* becomes negative and remains negative in the range of *Re* tested, demonstrating a drag reduction compared to the baseline cylinder. It is thought that an earlier transition to a critical *Re* of 5*.*0 × 10^4^, caused the wake region to be reduced, resulting in a negative *DI*^13^. At this critical *Re* of 5*.*0 × 10^4^, the microfiber-coated cylinder achieved a maximum drag reduction of 32% compared to that of the baseline cylinder. Once the *Re* increased beyond this critical value, the amount of drag reduction began to decrease.

For lower *Re*, the microfiber coating causes an increase in drag, indicated by the positive value for *DI*. This is due to the increase in surface roughness over the surface of the cylinder caused by the microfiber coating. If, as is generally the case for low *Re* flow, the boundary layer remains laminar over the entire surface, in increase in surface roughness will lead to an increase in skin friction and overall drag coefficient. However, at *Re* greater than 4.5 × 10^4^, the microfiber coating causes a sharp decrease in drag coefficient, indicated by the negative value for *DI*. This sharp drop is very similar to what is observed for the drag coefficient of a cylinder as *Re* increases past a certain critical *Re*. After the *Re* increases past a certain point, a phenomenon known as the drag crisis occurs, and with it a sharp drop in the drag coefficient. This critical *Re* is due to a transition in the boundary layer from laminar to turbulent, which causes a delay in flow separation and a reduction in pressure drag. The addition of the microfiber coating causes the laminar to turbulent transition at low *Re*, reducing the critical *Re* necessary for this drag crisis.

The performance of a fully microfiber-coated cylinder can also be compared against cylinders with different coverage of the microfiber coating. In previous studies, Hasegawa and Sakaue showed the drag reduction for different microfiber-coating configurations in terms of the coverage range and the location of the coating on a cylinder^39^*.* It was shown that a maximum drag reduction of 51%, which was higher than the drag reduction of the present study, is possible. This was achieved by applying strips of microfiber coating at 40^◦^ above and below the front stagnation point on the horizontal centerline at *Re* of 6*.*1 × 10^4^. By comparing this work to previous studies, it is shown that, at a given *Re*, there will be an optimum coverage and location to maximize drag reduction, and the fully microfiber-coated configuration is not optimal for maximizing the drag reduction.

## Conclusion

A fully microfiber-coated cylinder was studied for drag reduction at a range of Reynolds number *Re* in the subcritical-flow regime. A wake measurement was performed to determine the drag coefficient *c*_*d*_ as well as a characterization of the wake profile. The results demonstrated that *c*_*d*_ is a function of *Re* and the drag crisis occurred at *Re* ≈ 5 × 10^4^, instead of the critical *Re* of 3 × 10^5^ for a conventional smooth-surface cylinder. The maximum drag reduction of 32% was obtained compared to that of a smooth-surface cylinder at the same *Re*. Compared to a smooth-surface cylinder in the subcritical-flow regime, the fully covered microfiber coating functioned as a drag-reduction device in *Re* above 4*.*5 × 10^4^. It was shown that, at a given *Re*, there will be an optimum coverage and location to maximize drag reduction, and the fully microfiber-coated configuration is not optimal for maximizing the drag reduction.

## Data Availability

The datasets generated during and/or analyzed during the current study are available from the corresponding author on reasonable request.
